# Taurine Stimulates AMP-Activated Protein Kinase and Modulates the Skeletal Muscle Functions in Rats via the Induction of Intracellular Calcium Influx

**DOI:** 10.3390/ijms24044125

**Published:** 2023-02-18

**Authors:** Baojun Sun, Hitomi Maruta, Yun Ma, Hiromi Yamashita

**Affiliations:** 1 Graduate School of Health and Welfare Science, Okayama Prefectural University, Okayama 719-1197, Japan; 2 Department of Nutritional Science, Okayama Prefectural University, Okayama 719-1197, Japan

**Keywords:** taurine, skeletal muscle, AMPK, mitochondria, calcium

## Abstract

Taurine (2-aminoethanesulfonic acid) is a free amino acid abundantly found in mammalian tissues. Taurine plays a role in the maintenance of skeletal muscle functions and is associated with exercise capacity. However, the mechanism underlying taurine function in skeletal muscles has not yet been elucidated. In this study, to investigate the mechanism of taurine function in the skeletal muscles, the effects of short-term administration of a relatively low dose of taurine on the skeletal muscles of Sprague–Dawley rats and the underlying mechanism of taurine function in cultured L6 myotubes were investigated. The results obtained in this study in rats and L6 cells indicate that taurine modulates the skeletal muscle function by stimulating the expression of genes and proteins associated with mitochondrial and respiratory metabolism through the activation of AMP-activated protein kinase via the calcium signaling pathway.

## 1. Introduction

Taurine (2-aminoethanesulfonic acid) is a sulfur-containing free amino acid. In mammals, taurine is abundant in excitable tissues, particularly in the brain, retina, heart, and skeletal muscles [1,2,3]. Taurine is either obtained through diet, such as seaweed [4], seafood [5,6,7], and meat [8], or synthesized from cysteine in the body. Taurine functions in osmoregulation, cell membrane stabilization, anti-inflammatory effects, mitochondrial tRNA activities, and calcium homeostasis [2,3,9]. Taurine is essential for skeletal muscle function [10,11]. Taurine deficiency in taurine transporter knockout (taut−/−) mice reduced the skeletal muscle functions [12,13]. Taurine affects skeletal muscle contraction and enhances exercise performance by inhibiting oxidative stress in rats [14]. Taurine supplementation improves the electrical and contractile properties of skeletal muscle fibers in aged male Wistar rats [15]. In our previous study, long-term administration of taurine at a relatively low dose attenuates the age-related decline in O_2_ consumption and spontaneous locomotor activity with the activation of AMP-activated protein kinase (AMPK) [16]. AMPK is a heterotrimeric protein kinase [17]. It is a sensor of cellular energy status that plays a key role in the regulation of energy metabolism, oxidative capacity, and exercise capacity by phosphorylating key metabolic enzymes in both biosynthetic and oxidative pathways [18,19,20]. Taurine plays a beneficial role in enhancing the performance and duration of exercise. Yatabe et al. showed that the administration of taurine (500 mg/kg/d) to Sprague–Dawley (SD) rats for two weeks maintained the taurine levels in skeletal muscles during exercise and upregulated the physical endurance [21]. Miyazaki et al. showed that the oral administration of taurine (20, 100, and 500 mg/kg/d) to SD rats for two weeks improved their exercise performance [14]. Dawson et al. demonstrated that the administration of 3% taurine in drinking water for 1 month enhanced exercise performance in rats [22]. They suggested that taurine might be involved in enhancing the skeletal muscle contractile mechanism and mitigating the oxidative damage associated with exercise [22]. However, only a few studies have described how taurine levels change in skeletal muscles after oral administration and its specific roles in skeletal muscle function. Taurine may have an important role in muscle function and may control muscle metabolism and gene expression; however, the specific action mechanisms remain unclear [23].

In this study, we investigated the changes in taurine levels in skeletal muscles and blood after oral administration, the beneficial roles of taurine, and its mechanism of action on skeletal muscle functions in SD rats and L6 myotubes. Here, we show that taurine modulates the expression of genes and proteins associated with mitochondrial and respiratory metabolism and skeletal muscle function through the activation of AMPK via the calcium signaling pathway.

## 2. Results

### 2.1. Effects of Taurine Administration on Taurine Levels in the Plasma and Skeletal Muscles of SD Rats

**Figure 1:**
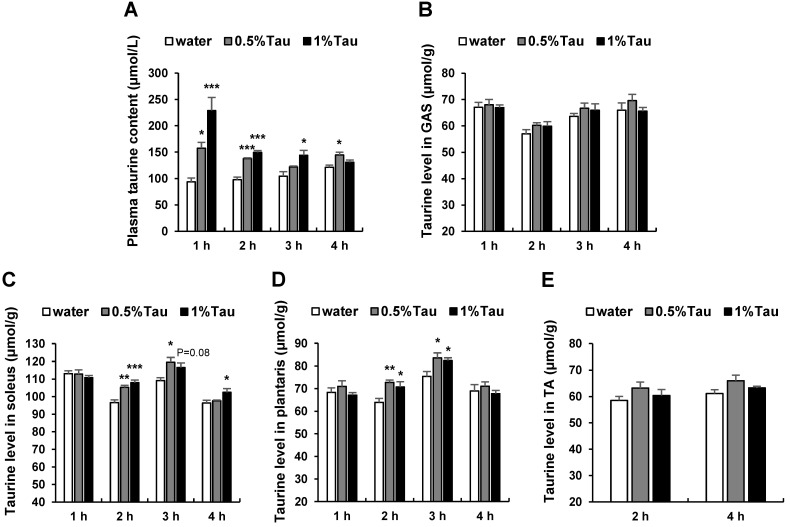
Effects of short-term taurine supplementation on taurine levels in the plasma and skeletal muscles of rats. Taurine (Tau) groups of 0.5% and 1% were orally administered taurine at 0.5% (25 mg taurine /kg body weight [BW]) and 1% (50 mg taurine/kg BW), respectively, for 10 d, and the taurine levels in plasma and skeletal muscles were measured 1–4 h after the administration of taurine on the 10th day in rats at 14 weeks of age. (**A**) Taurine level in plasma (μmol/L). (**B**) Taurine level in gastrocnemius (GAS) muscle (μmol/g). (**C**) Taurine level in soleus muscle (μmol/g). (**D**) Taurine level in plantaris muscle (μmol/g). (**E**) Taurine level in the tibialis anterior (TA) muscle (μmol/g). Values shown represent the mean ± standard error (SE) (n = 3–6). * *p* < 0.05, ** *p* < 0.01, *** *p* < 0.001, statistically significant vs. the value of water group. Results were analyzed using Dunnett’s test.

Previously, we investigated the effect of long-term taurine supplementation on age-related changes in skeletal muscle function and found that long-term taurine supplementation at a relatively low dose modulates age-related changes in respiration, metabolism, and skeletal muscle function. To investigate the function of taurine in skeletal muscles in more detail, changes in taurine concentration in plasma and skeletal muscles were measured from 1 to 4 h after the oral administration of taurine to rats at 14 weeks of age. Plasma taurine concentration was significantly increased at 1 h after the administration of taurine in a dose-dependent manner compared to that in the water group (Figure 1A). Taurine concentrations in the soleus muscle of both taurine groups were significantly increased at 2 h, and those in the 0.5% taurine group at 3 h and in the 1% taurine group at 4 h were significantly increased compared to those in the water group (Figure 1C). Taurine concentrations in the plantaris muscle of both taurine groups were significantly increased at 2 and 3 h compared to those in the water group (Figure 1D), while taurine concentrations in the gastrocnemius (GAS) muscle of both taurine groups showed a tendency to increase, but not significantly, at 2 and 3 h compared to those in the water group (Figure 1B). Taurine concentrations in the tibialis anterior (TA) muscle of both taurine groups showed a tendency to increase, but not significantly, at 2 and 4 h compared to those in the water group (Figure 1E).

### 2.2. Effects of Taurine on the Expression Levels of Myogenic Genes in the Skeletal Muscles of SD Rats

**Figure 2:**
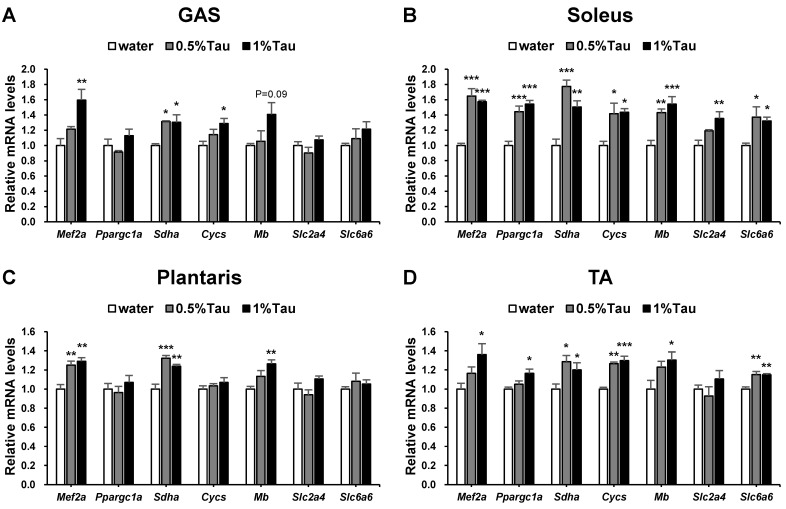
Effects of short-term taurine supplementation on the relative mRNA expression levels of skeletal muscle function-related genes in GAS (**A**), soleus (**B**), plantaris (**C**), and TA (**D**) muscles of rats in the water, 0.5% taurine, and 1% taurine groups. Skeletal muscles of SD rats at 14 weeks of age were collected 4 h after administration on the 10th day and analyzed via quantitative reverse transcription-polymerase chain reaction (qRT-PCR) to determine the mRNA expression levels of myocyte enhancer factor 2A (*Mef2a*/ MEF2A), peroxisome proliferator-activated receptor gamma coactivator 1-alpha (*Ppargc1a*/PGC-1α), succinate dehydrogenase complex flavoprotein subunit A (*Sdha*/SDH), cytochrome c, somatic (*Cycs*/Cycs), myoglobin (*Mb*), solute carrier family 2 member 4 (*Slc2a4*/GLUT4), and solute carrier family 6 member 6 (*Slc6a6*/TauT). The administration levels of taurine are indicated in Figure 1. Values shown represent the mean ± SE (n = 3–6). * *p* < 0.05, ** *p* < 0.01, *** *p* <0.001, statistically significant versus the value of the water group. Results were analyzed using Dunnett’s test.

The effects of relatively low-dose and short-term taurine (10 days) supplementation on the expression of genes associated with skeletal muscle function were analyzed at 4 h after taurine administration on the 10th day. Expression of the myocyte enhancer factor 2A (*Mef2a*/MEF2A) and cytochrome c, somatic (*Cycs*/Cycs) genes was significantly increased in the GAS muscle of the 1% taurine group, and the succinate dehydrogenase complex flavoprotein subunit A (*Sdha*/SDH) gene was increased in the GAS muscle of both taurine groups compared to that in the water group (Figure 2A). In the soleus muscle, MEF2A, peroxisome proliferator-activated receptor gamma coactivator 1-alpha (*Ppargc1a*/PGC-1α), SDH, Cycs, myoglobin (*Mb*), and solute carrier family 6 member 6 (*Slc6a6*/TauT) genes were increased in both taurine groups, and solute carrier family 2 member 4 (*Slc2a4*/GLUT4) gene expression was increased in the 1% taurine group compared to that in the water group (Figure 2B). In the plantaris, MEF2A and SDH genes were increased in both taurine groups, and the myoglobin gene was increased in the 1% taurine group compared to that in the water group (Figure 2C). In the TA muscle, SDH, Cycs, and TauT genes were increased in both taurine groups, and MEF2A, PGC-1α, and myoglobin genes were higher in the 1% taurine group than in the water group (Figure 2D).

### 2.3. Effects of Taurine on the Phosphorylation of AMPK and the Expression Levels of Myogenic Proteins

**Figure 3:**
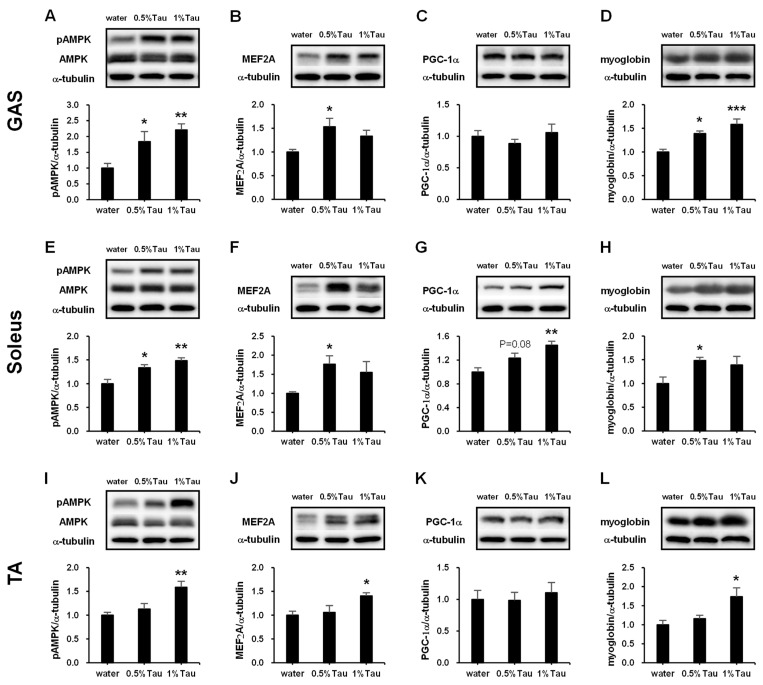
Effects of short-term taurine supplementation on the phosphorylation of AMP-activated protein kinase (AMPK) (**A**,**E**,**I**) and expression levels of MEF2A (**B**,**F**,**J**), PGC-1α (**C**,**G**,**K**), and myoglobin (**D**,**H**,**L**) proteins in GAS, soleus, and TA muscles of rats in the water, 0.5% taurine, and 1% taurine groups. Skeletal muscles of SD rats at 14 weeks of age were collected 4 h after administration on the 10th day. The administration levels of taurine are indicated in Figure 1. Proteins were extracted and analyzed by Western blot as described in the Materials and Methods section. Values shown represent the mean ± SE (n = 3–6). * *p* < 0.05, ** *p* < 0.01, *** *p* < 0.001, statistically significant vs. the values of the water group. Results were analyzed using Dunnett’s test.

AMPK is a key mediator of cell signaling pathways intrinsically linked to muscle function and metabolism [24]. To determine whether the function of taurine is associated with AMPK phosphorylation in the skeletal muscle of SD rats at 14 weeks of age, phosphorylated AMPK was analyzed in skeletal muscles after taurine administration. In the GAS and soleus muscles, phosphorylated AMPK was significantly increased in both taurine groups compared to that in the water group (Figure 3A,E). In the TA muscle, phosphorylated AMPK was increased in the 1% taurine group compared to that in the water group (Figure 3I). MEF2A, PGC-1α, and myoglobin proteins, which are regulated by AMPK activation [25,26,27,28], were also analyzed. MEF2A protein was increased in the GAS muscle in the 0.5% taurine group compared to that in the water group (Figure 3B). Myoglobin was significantly increased in the GAS muscle of both taurine groups compared to that in the water group (Figure 3D). In the soleus muscle, MEF2A and myoglobin levels were significantly higher in the 0.5% taurine group (Figure 3F,H), and PGC-1α protein expression was significantly higher in the 1% taurine group (Figure 3G) than in the water group. In the TA muscle, the expression of MEF2A and myoglobin proteins was higher in the 1% taurine group than in the water group (Figure 3J,L). In the plantaris muscle, the phosphorylation of AMPK and expression of MEF2A and myoglobin proteins tended to increase in both taurine groups (not shown).

### 2.4. Effects of Taurine Administration on Mitochondrial DNA (mtDNA) and SDH Staining in the Skeletal Muscles of SD Rats

**Figure 4:**
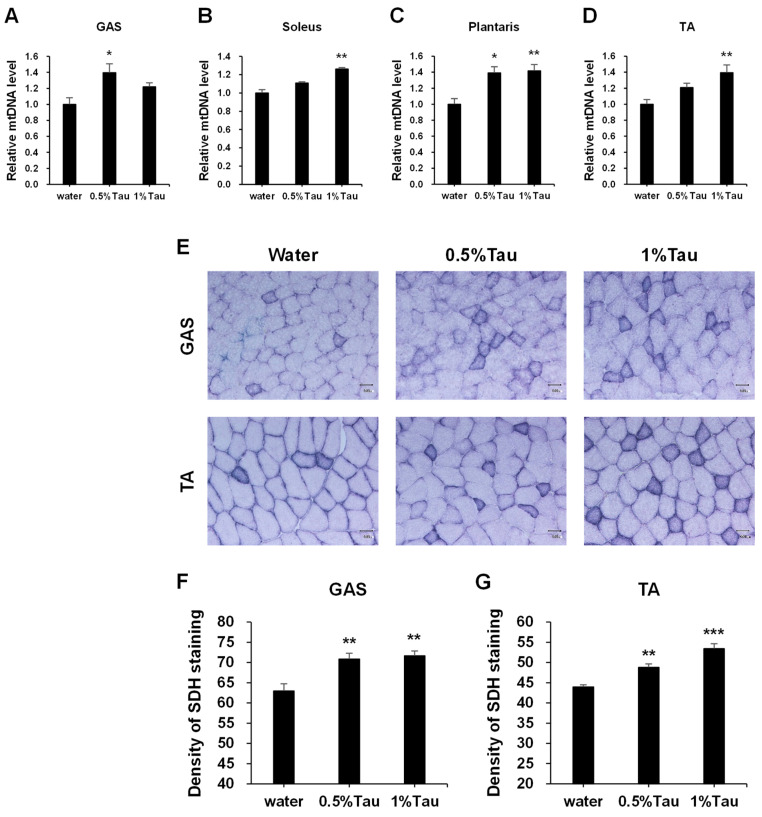
Effects of short-term taurine supplementation on the mitochondrial DNA (mtDNA) content (**A**–**D**) in the GAS, soleus, plantaris, and TA muscles, and SDH staining (**E**) and ImageJ (Ver. 1.52p) analysis of SDH staining (**F**,**G**) in the GAS and TA muscles of rats in the water, 0.5% taurine, and 1% taurine groups. Skeletal muscles of SD rats at 14 weeks of age were collected 4 h after administration on the 10th day. The administration levels of taurine are indicated in Figure 1. Scale bar, 500 μm. Values shown represent the mean ± SE (n = 3–6). * *p* < 0.05, ** *p* < 0.01, *** *p* < 0.001, statistically significant vs. the values of water group. Results were analyzed using Dunnett’s test.

To examine the effect of taurine supplementation on mitochondrial proliferation, the relative quantity of mtDNA in the GAS, soleus, plantaris, and TA muscles and the staining level of SDH in the GAS and TA muscles were analyzed. SDH staining was performed to characterize mitochondrial enzyme function and myofiber oxidative capacity [29,30,31]. Significantly higher quantities of mtDNA were observed in the GAS of the 0.5% taurine group, in the soleus and TA of the 1% taurine group, and in the plantaris of both taurine groups than those in the water group (Figure 4A–D). The SDH staining level was significantly higher in the Gas and TA muscles in both taurine groups than in the water group (Figure 4F,G).

### 2.5. Effects of Taurine on the Expression Levels of Myogenic Genes in L6 Cells

**Figure 5:**
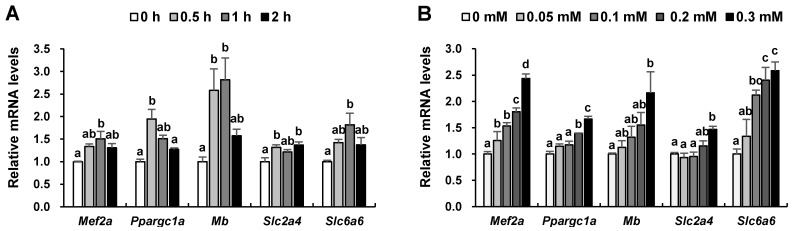
Effects of taurine on the expression levels of MEF2A, PGC-1α, myoglobin, GLUT4, and TauT genes in L6 myotubes. (**A**) L6 myotubes were treated with 0.3 mM taurine for the time periods indicated. (**B**) L6 cells were treated with 0, 0.05, 0.1, 0.2, and 0.3 mM taurine for 1 h. Total RNA was extracted from the cells after treatment with taurine and qRT-PCR analysis was carried out to determine the mRNA expression levels of *Mef2a*, *Ppargc1a*, *Mb*, *Slc2a4*, and *Slc6a6* in L6 cells, as described in the Materials and Methods section. Values shown represent the mean ± SE (n = 3–6). Results were analyzed using one-way analysis of variance (ANOVA) followed by Tukey’s test for multiple comparisons. Values with different superscript lowercase letters indicate significant differences (*p* < 0.05).

To clarify the effect of taurine on skeletal muscle function, the induction of genes and proteins associated with muscle function in L6 myotubes was analyzed. A time-course experiment revealed that the expression of MEF2A, PGC-1α, myoglobin, GLUT4, and TauT genes was induced significantly after 0.5 h and/or 1 h with taurine treatment (0.3 mM compared with those of the non-treated control (Figure 5A)). L6 cells were treated with different concentrations of taurine (0, 0.05, 0.1, 0.2, and 0.3 mM) for 1 h. The expression levels of the MEF2A gene were significantly increased after treatment with taurine (0.05–0.3 mM) compared to those in the non-treated group (Figure 5B). The expression of the PGC-1α gene was significantly increased with taurine at 0.2 and 0.3 mM, the TauT gene was significantly increased from 0.1 to 0.3 mM, and the myoglobin and GLUT4 genes were significantly increased with taurine at 0.3 mM compared to the non-treated group (Figure 5B).

### 2.6. Taurine Induces the Phosphorylation of AMPK in L6 Myotubes

**Figure 6:**
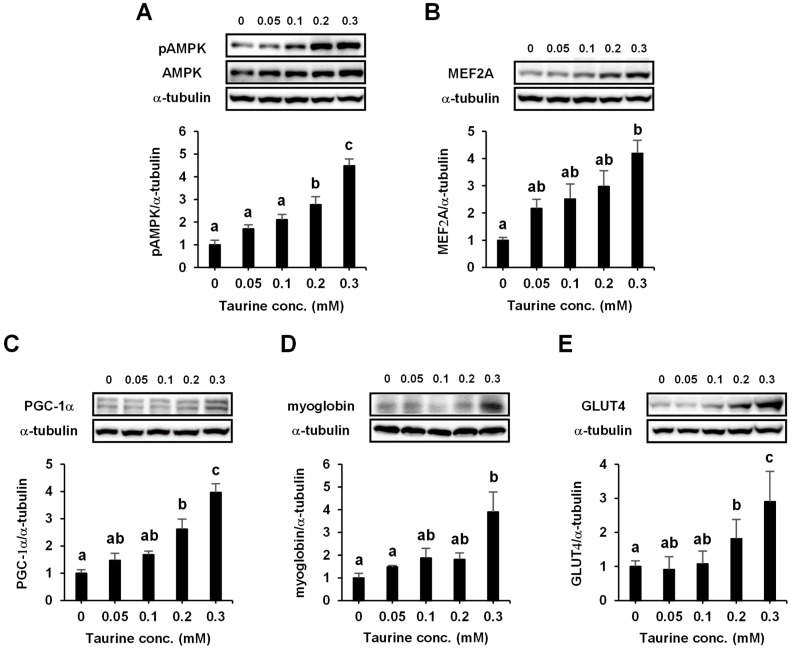
Taurine stimulates the phosphorylation of AMPK (**A**) and the expression of MEF2A (**B**), PGC-1α (**C**), myoglobin (**D**), and GLUT4 (**E**) proteins in L6 myotubes. L6 myotubes were treated with 0, 0.05, 0.1, 0.2, and 0.3 mM taurine for 1 h. After treatment, L6 cells were analyzed by Western blot, as described in Materials and Methods section. Values shown represent the mean ± SE (n = 3–6). Results were analyzed using one-way ANOVA followed by Tukey’s test for multiple comparisons. Values with different superscript lowercase letters indicate significant differences (*p* < 0.05).

To determine whether treatment with taurine could induce the phosphorylation of AMPK, the change in phosphorylated AMPK levels was analyzed. Phosphorylated AMPK levels were significantly increased by taurine treatment (0.2 and 0.3 mM) compared to the untreated control (Figure 6A). The expression levels of MEF2A, PGC-1α, myoglobin, and GLUT4 proteins, which are regulated by the activation of AMPK [25,26,27,28], were significantly increased with 0.2 and 0.3 mM taurine (Figure 6B–E).

### 2.7. Effects of Taurine Transporter Antagonist, Guanidinoethyl Sulfonate (GES), and AMPK Inhibitor, Adenine 9-β-D-arabinofuranoside (araA), on the Phosphorylation of AMPK and Expression Levels of Myogenic Genes and Proteins

**Figure 7:**
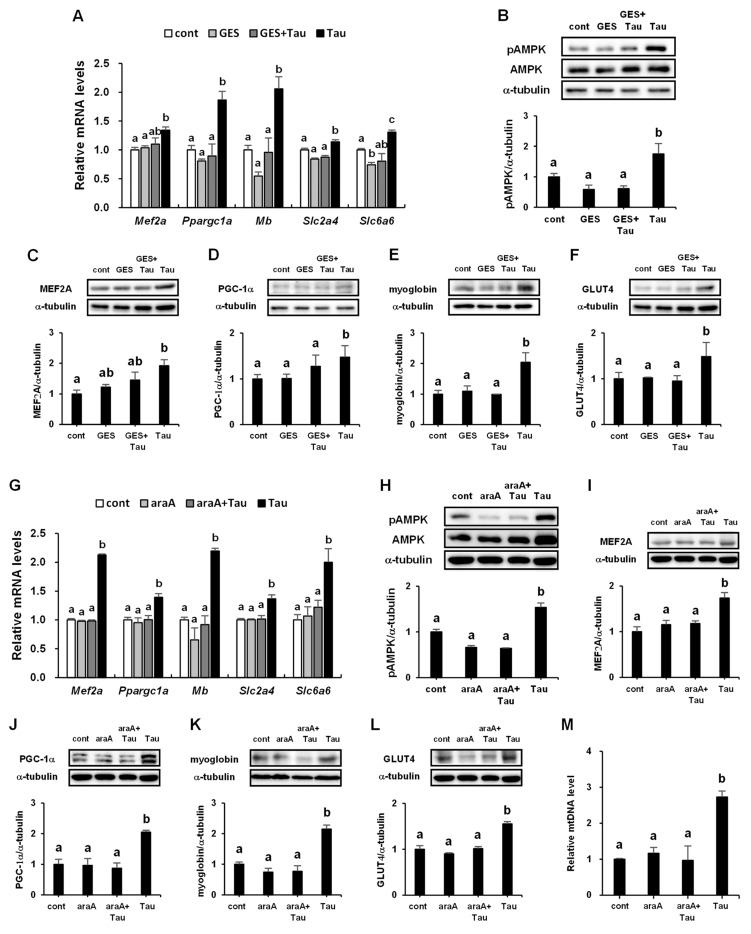
Effects of taurine transporter antagonist, guanidinoethyl sulfonate (GES), and AMPK inhibitor, adenine 9-β-D-arabinofuranoside (araA), on the stimulated phosphorylation of AMPK and expression levels of myogenic genes and proteins after treatment with taurine in L6 myotubes. L6 cells were treated with 0.3 mM taurine for 1 h in the presence or absence of 0.3 mM GES preincubated for 1 h (**A**–**F**) or 2 mM araA preincubated for 20 min (**G**–**M**) before treatment with taurine. After treatment, L6 cells were analyzed via qRT-PCR analysis (**A**,**G**,**M**) or Western blot (**B**–**F**,**H**–**L**), as described in the Materials and Methods section. Values shown represent the mean ± SE (n = 3–6). Results were analyzed using one-way ANOVA followed by Tukey’s test for multiple comparisons. Values with different superscript lowercase letters indicate significant differences (*p* < 0.05).

To further investigate the signaling pathway of taurine, the roles of taurine transporters and AMPK were examined. The taurine transporter antagonist, guanidinoethyl sulfonate (GES) [32,33], suppressed the expression of PGC-1α, myoglobin, GLUT4, and TauT genes, which were induced by taurine treatment (Figure 7A). The expression of the MEF2A gene induced by taurine treatment tended to be decreased by GES treatment. GES treatment also suppressed the phosphorylation of AMPK (Figure 7B) and expression of PGC-1α, myoglobin, and GLUT4 proteins, which were induced by taurine treatment (Figure 7D–F). The expression of the MEF2A protein induced by taurine treatment tended to be decreased by GES treatment (Figure 7C). Treatment of taurine with the AMPK inhibitor, adenine 9-β-D-arabinofuranoside (araA) [34], decreased the phosphorylation of AMPK (Figure 7H) and suppressed the expression of MEF2A, PGC-1α, myolobin, GLUT4, and TauT genes induced by taurine treatment (Figure 7G). Treatment with araA suppressed the taurine-induced expression of MEF2A, PGC1-α, myoglobin, and GLUT4 proteins (Figure 7I–L). Taurine treatment increased the level of mtDNA, but it was suppressed in the presence of araA (Figure 7M).

### 2.8. Induction of Intracellular Calcium Influx after Treatment with Taurine in L6 Cells

**Figure 8:**
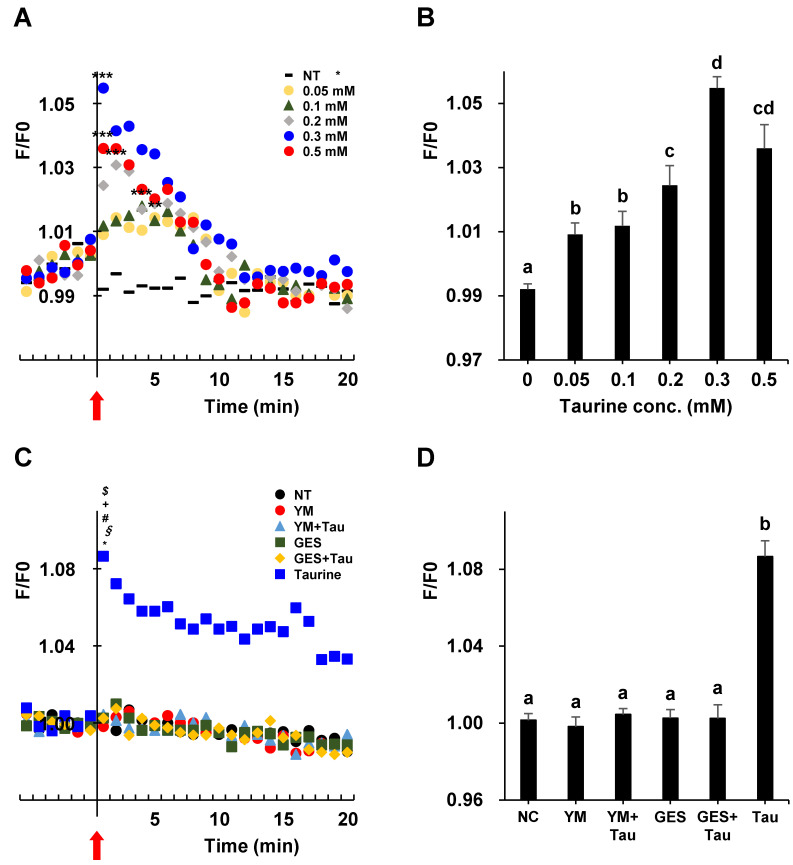
Taurine induces [Ca^2+^]i influx in L6 myotubes. (**A**) Changes in the [Ca^2+^]i influx were measured in response to 0.05–0.5 mM taurine treatment in L6 cells. Arrow indicates the addition of taurine. NT: non-treatment condition. The data are the average values of 3–12 independent experiments. Results were analyzed using one-way ANOVA followed by Tukey’s test for multiple comparisons. Statistical differences are shown as ** *p* < 0.05, *** *p* < 0.001, compared to NT. (**B**) Peaks of average values were analyzed using one-way ANOVA followed by Tukey’s test for multiple comparisons. Values with different superscript lowercase letters indicate significant differences (*p* < 0.05). (**C**) Changes in [Ca^2+^]i influx were measured in response to 0.3 mM taurine in L6 cells treated or not treated with YM-254890 (YM, 1.0 μM) or GES (0.3 mM) for 24 h. Arrow indicates the addition of taurine. NT: non-treatment condition. The data are the average values of 3–12 independent experiments. The results were analyzed using one-way ANOVA followed by Tukey’s test for multiple comparisons. Statistical differences are shown as * *p* < 0.001, compared with NT. § *p* < 0.001, compared with YM. # *p* < 0.001, compared with YM + taurine. + *p* < 0.001, compared with GES. $ *p* < 0.001, compared with GES + taurine. (**D**) Peaks of average values were analyzed using one-way ANOVA, followed by Tukey’s test for multiple comparisons. Values with different superscript lowercase letters indicate significant differences (*p* < 0.05).

AMPK is activated by calcium/calmodulin-dependent protein kinase kinase (CaMKK), which is activated by the intracellular [Ca^2+^]i influx [35,36,37]. We focused on the calcium influx and its signaling pathways. To determine whether taurine could stimulate calcium influx in L6 myotubes, changes in calcium levels were measured. The treatment of taurine stimulated calcium influx in a dose-dependent manner from 0.05 to 0.3 mM (Figure 8A,B). The phospholipase C (PLC) is a class of membrane-associated enzymes that is involved in the regulation of Ca^2+^ [38]. To examine the involvement of PLC activation in calcium influx [38] by taurine, the PLC inhibitor YM-254890 (YM) [39] was used. YM suppressed taurine-induced intracellular calcium influx (Figure 8C,D). The taurine transporter antagonist, GES, also suppressed the intracellular calcium influx induced by taurine (Figure 8C,D).

### 2.9. Effects of PLC Inhibitor on AMPK Phosphorylation and Myogenic Gene and Protein Expressions

**Figure 9:**
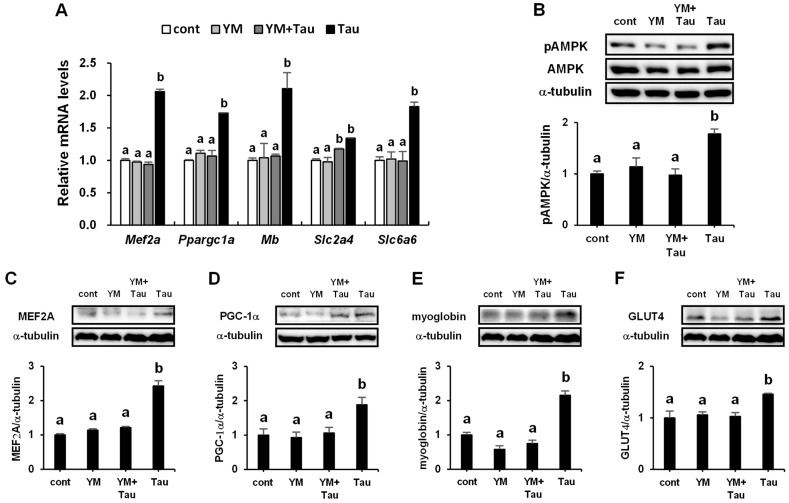
Phospholipase C (PLC) inhibitor suppresses the phosphorylation of AMPK and the expression levels of MEF2A, PGC-1α, myoglobin, and GLUT4 genes and proteins. L6 cells were treated with 0.3 mM taurine for 1 h in the presence or absence of 1.0 μM YM and preincubated for 5 min. After treatment, L6 cells were analyzed via qRT-PCR analysis (**A**) or Western blot (**B**–**F**), as described in the Materials and Methods section. Values shown represent the mean ± SE (n = 3–6). The results were analyzed using one-way ANOVA followed by Tukey’s test for multiple comparisons. Values with different superscript lowercase letters indicate significant differences (*p* < 0.05).

To examine the involvement of PLC in taurine-induced calcium signaling, the effects of YM on the phosphorylation of AMPK and myogenic genes and proteins were analyzed. YM suppressed the induced expression of MEF2A, PGC-1α, myoglobin, GLUT4, and TauT genes following treatment with taurine (Figure 9A). Similarly, treatment with YM reduced the induction of AMPK phosphorylation and the expression of MEF2A, PGC-1α, myoglobin, and GLUT4 proteins induced by taurine treatment (Figure 9B–F).

## 3. Discussion

Taurine is present in the free form in skeletal muscle and is essential for skeletal muscle function [10,23]. Numerous studies have shown that taurine supplementation can improve skeletal muscle function; however, the molecular mechanism underlying the action of taurine on skeletal muscle function remains unclear. Here, we investigated the effect of taurine on skeletal muscle function in experimental animals and cultured L6 myotubes. Plasma taurine concentration increased significantly, peaked at 1 h, and then declined (Figure 1A). The concentration of taurine in the soleus and plantaris muscles increased significantly from 2 to 3 h (Figure 1C,D). Taurine may be absorbed into the blood after administration and then transported to the skeletal muscles within 2 h. The taurine content in skeletal muscles may return to basal levels 4 h after administration. Taurine concentrations in the GAS and TA muscles of both taurine groups showed a tendency to increase, but not statistically significantly. Sved et al. [40] reported the concentration of taurine in plasma and tissues after dosing ^14^C-taurine in rats. They describe that the rate of elimination of intracellular taurine will depend on the rate of turnover of the intracellular pool for that particular tissue. The taurine level in the muscles may depend on taurine absorption and processing capacity.

The expression levels of myogenic genes associated with mitochondrial and respiratory metabolism were significantly increased in the skeletal muscles of rats administered taurine compared to those in the control group. The phosphorylation of AMPK in the GAS, soleus, and TA muscles of rats administered taurine was significantly increased compared to that in rats in the control group (Figure 3A,E,I). This suggests that the effect of taurine on the muscles is independent of the level of taurine in the muscles. AMPK is a sensor of cellular energy status and plays a key role in the regulation of energy metabolism, oxidative capacity, and exercise capacity [41,42]. Activation of AMPK can increase mitochondrial enzymes in skeletal muscles [43]. MEF2A is a member of the MEF2 family of transcription factors involved in skeletal muscle differentiation and is regulated by AMPK [25,28]. The expression levels of the MEF2A gene were significantly increased in the four skeletal muscles of the 0.5% and 1% taurine groups (Figure 2). The expression levels of the MEF2A protein were also increased in the GAS, soleus, and TA muscles of the 0.5% and 1% taurine groups (Figure 3B,F,J). Both MEF2 and AMPK are involved in the regulation of GLUT4 gene transcription [44,45]. GLUT4 is a glucose transporter protein [46]. The expression of the GLUT4 gene was significantly increased in the soleus muscle of the 1% taurine group (Figure 2B). The PGC-1α gene and protein expressions in the soleus muscle were significantly increased in the 1% taurine group (Figure 2B and 3G). PGC-1α is a transcriptional coactivator that plays a key role in the regulation of mitochondrial biogenesis and oxidative metabolism [47,48] and its activity is regulated by AMPK [49,50]. The mRNA expression levels of SDH, which is a marker enzyme of mitochondria [29,30,31], were significantly increased in the four kinds of skeletal muscles of both taurine groups 4 h after the administration of taurine compared to those in the control group (Figure 2). SDH staining levels of the GAS and TA muscles in both taurine groups were significantly higher than those in the control group (Figure 4F,G). The gene expression levels of Cycs, which is a component of the electron transport chain of mitochondria, a marker of mitochondrial biogenesis [51], were also higher in the GAS, soleus, and TA muscles of the 0.5% and 1% taurine groups than in the control group (Figure 2A,B,D). In addition, MEF2A and PGC-1α are also involved in the expression of myoglobin, which is an essential oxygen storage hemoprotein that facilitates oxygen transport and is required for lipid and glucose oxidation within skeletal muscles [26]. The expression levels of the myoglobin gene were significantly increased in the soleus, plantaris, and TA muscles of the 0.5% and 1% taurine groups compared to those in the control group (Figure 2B–D). The expression levels of myoglobin protein were significantly increased in the GAS, soleus, and TA muscles of the 0.5% and 1% taurine groups compared to those in the control group (Figure 3D,H,L). These results suggest that taurine supplementation increases mitochondrial biogenesis and improves oxidation capacity associated with skeletal muscle function through these molecules regulated by the activation of AMPK.

To further investigate the mechanism of action of taurine on skeletal muscle function, experiments were conducted using L6 cells. Treatment with taurine (0.3 mM), which is near the plasma taurine level, increased the expression levels of myogenic genes associated with mitochondrial function and respiratory metabolism, as seen in the skeletal muscles of SD rats (Figure 5A,B). Taurine treatment stimulated the phosphorylation of AMPK and increased the expression levels of MEF2A, PGC-1α, myoglobin, and GLUT4 proteins (Figure 6A–E), while in the GAS and TA muscles, expression of the PGC-1α protein was not increased significantly. Activated AMPK functions not only in the induction of the PGC-1α gene [49] but also in the phosphorylation of PGC-1α [27]. Iwabu et al. reported that PGC-1α is activated by AMPK via phosphorylation and by deacetylation through SIRT1 activation [36]. This suggests that there may be different mechanisms of action of AMPK, which cooperates with or without SIRT1, for the activation of PGC-1α among muscles and cells. To elucidate the signaling pathway of taurine, we used antagonists of the taurine transporter, GES [32,33], the AMPK inhibitor, araA [34], and the PLC inhibitor, YM [39]. The enhancing effects of taurine on the phosphorylation of AMPK and the expression of myogenic genes and proteins were completely suppressed by GES treatment (Figure 7A–F). This suggests that taurine performs its physiological function when it enters the cells through the taurine transporter. As for the treatment with the AMPK inhibitor araA, the effects of taurine on the phosphorylation of AMPK and on the expression of myogenic genes and proteins were also suppressed (Figure 7G–L). This indicates that taurine performs its physiological function by activating AMPK and stimulating its downstream factors, MEF2A, PGC-1α, myoglobin, and GLUT4. Taurine treatment stimulated calcium influx, but the PLC inhibitor, YM, inhibited this stimulation (Figure 8A–D). In addition, after treatment with YM, the effects of taurine on the phosphorylation of AMPK and the expression of myogenic genes and proteins were completely suppressed (Figure 9A–F). Activated PLC increases the inositol-1,4,5-triphosphate levels and induces calcium influx in cells [38,52]. This indicates that taurine can stimulate calcium influx via PLC, and Ca^2+^ can activate AMPK by activating CaMKK [35,52]. However, further studies will be needed to determine whether TauT is coupled with G-protein and the mechanism by which taurine associates with PLC.

Collectively, this study demonstrates that taurine can stimulate PLC to increase the calcium influx in the cells via the interaction with the taurine transporter, thereby activating AMPK. Through the PLC–Ca^2+^–AMPK signaling pathway, the expression levels of genes and proteins associated with the key factors, MEF2A and PGC-1α, are increased, along with the expression levels of GLUT4, myoglobin, and mitochondrial proteins, SDH and Cycs. Our findings provide insights into the role of taurine in improving skeletal muscle function.

## 4. Materials and Methods

### 4.1. Materials

Taurine, perchloric acid, trichloroacetic acid, acetonitrile, sodium tetraborate, o-phthalaldehyde (OPA), 2-mercaptoethanol, formalin, and α-tubulin antibodies were purchased from FUJIFILM Wako Pure Chemical Industries Ltd. (Osaka, Japan). AMPK inhibitor, araA, nitro blue tetrazolium, and Dulbecco’s modified Eagle’s medium (DMEM) were purchased from Sigma-Aldrich (St. Louis, MO, USA). Rat L6 myoblasts (JCRB9081) were purchased from JCRB Cell Bank (Osaka, Japan). Fetal bovine serum (FBS) and 0.02% ethylenediaminetetraacetic acid (EDTA) were purchased from MP Biomedicals (Santa Ana, CA, USA), while penicillin, streptomycin, and 0.25% trypsin were purchased from Invitrogen (Carlsbad, CA, USA). Mount Quick was obtained from DAIDO SANGYO Co., Ltd. (Tokyo, Japan). Sepasol-RNA I Super G, sodium succinate, and Bullet Blocking One were purchased from Nacalai Tesque (Kyoto, Japan). RNase inhibitor, ReverTra Ace qPCR Master Mix, and gDNA remover kit were purchased from Toyobo Co., Ltd. (Osaka, Japan). KAPA SYBR FAST qPCR kit was purchased from Kapa Biosystems (Wilmington, MA, USA). Antibodies against AMPKα and phosphorylated AMPKα were purchased from Cell Signaling Technology (Danvers, MA, USA). Antibodies against peroxisome proliferator-activated receptor gamma coactivator 1-alpha (PGC-1α), myocyte enhancer factor 2A (MEF2A), and myoglobin were purchased from Santa Cruz Biotechnology (Dallas, TX, USA). The taurine transport antagonist, GES, was purchased from Cayman Chemical (Ann Arbor, MI, USA). PLC inhibitor, YM, and α-tubulin antibodies were purchased from FUJIFILM Wako Pure Chemical Corporation (Osaka, Japan).

### 4.2. Animal Experiments

All animal experiments were performed in accordance with the guidelines of the Okayama Prefectural University and the laws and notifications of the Japanese government. All animal experiments were approved by the Animal Care and Use Committee of the Okayama Prefectural University (protocol number 3-3). Male SD rats at 11 weeks of age were purchased from Charles River Laboratories Japan Inc. (Yokohama, Japan). Animals were housed individually in an air-conditioned room at 25 °C with an alternating 12 h light and dark cycle (light, 8:00–20:00). The animals had free access to commercial food (CE-2; CLEA Japan, Inc., Tokyo, Japan) and water. The food intake and body weight were measured daily.

At 11 weeks of age, the rats were randomly assigned to one of the following treatment groups: water (control), 0.5% taurine (*w/v*), and 1% taurine (*w/v*) groups. The rats were fed a normal laboratory diet for two weeks for acclimatization and administered their respective doses from 13 weeks for 10 consecutive days. The administered dose of taurine was 25 mg/kg body weight (BW) in the 0.5% taurine group and 50 mg/kg BW in the 1% taurine group. Dissection was performed 1–4 h after administration on the 10th day. Blood and tissue samples were also collected. Blood samples were collected in heparinized tubes and centrifuged at 3000 rpm at 4 °C for 15 min to obtain plasma. Portions of tissues were frozen in liquid nitrogen and stored at −80 °C until subsequent measurements. Other tissues were isolated, cramped, and lyophilized (TOKYO RIKAKIKAI, Tokyo, Japan) for the measurement of taurine levels.

### 4.3. Culture of L6 Cells

Rat L6 cells, medium, and other reagents used for cell culture were as previously described [25,39]. L6 myoblasts were grown in DMEM containing 10% (*v/v*) FBS, 100 units/mL penicillin, and 100 μg/mL streptomycin in 5% CO_2_ at 37 °C. For myotube differentiation, the medium was changed to DMEM containing 2% (*v/v*) horse serum when the myoblasts were 80% confluent. Myotubes were harvested 8–11 d after differentiation and the experimental procedures were initiated. Differentiated myotubes were incubated with inhibitors with or without taurine.

### 4.4. Biochemical Analysis

Biochemical analysis was performed as previously described [16]. Freeze-dried tissue samples were homogenized with 2 mL of 0.5 N perchloric acid and centrifuged. The supernatant was neutralized with 5 N KOH and collected in separate tubes after centrifugation. Trichloroacetic acid (10%, *v/v*) was added to the plasma to remove the plasma proteins. The suspension was shaken for 1 h, centrifuged, and the supernatant was neutralized with 1 N KOH and collected. Taurine was derivatized with OPA derivatization reagent containing 200 μL of 25 mg/mL OPA in ethanol, 10 μL of 2-mercaptoethanol, and 2.5 mL of 0.1 M sodium tetraborate buffer (pH 9.5) and measured as a fluorescent adduct using a high-performance liquid chromatography (HPLC) system. The HPLC system (Shimadzu Corp., Tokyo, Japan) with LC-20AB HPLC pumps, a CTO-20A column oven, an SPD-M20A detector, and a reverse-phase column (Shim Pack VP-ODS separation, 250 L × 4.6; Shimadzu Corp., Tokyo, Japan) were employed. The flow rate was 1 mL/min, injection volume was 5 μL, wavelength for detection was 338 nm, and the column temperature was 40 °C using phosphate buffer mixed with acetonitrile (70:30) as the mobile phase. Taurine was derivatized by mixing with an equal volume of the OPA-derivatizing reagent for 1 min before injecting the reaction mixture into the column.

### 4.5. Histological Analysis

GAS muscle and TA muscle tissues were sliced into 10 μm sections using a Leica CM3050 S cryostat (Leica Microsystems, Wetzlar, Germany) at −20 °C. Tissue sections were air-dried at 20–25 °C for 5 min and incubated in 50 mM phosphate buffer containing 50 mM sodium succinate and 0.5 mg/mL nitro blue tetrazolium at 37 °C for 40 min [16,53]. The sections were briefly washed thrice with distilled water and mounted using the Mount-Quick aqueous mounting medium. Images were captured with a CCD camera (Olympus Optical, Tokyo, Japan) at a magnification of ×100.

### 4.6. Quantitative Reverse Transcription-Polymerase Chain Reaction (qRT-PCR) Analysis

Total RNA was extracted from frozen tissue samples using Sepasol-RNA I Super G. Genomic DNA was isolated using an extraction buffer containing 4 M guanidine thiocyanate, 50 mM sodium citrate, and 1 M Tris. An RNase inhibitor was added according to the manufacturer’s instructions. Total RNA was quantified, and cDNA was prepared using ReverTra Ace qPCR Master Mix and a gDNA remover kit. Quantitative Reverse Transcription-Polymerase Chain Reaction (qRT-PCR) was performed on a StepOnePlus detection system (Applied Biosystems, Foster City, CA, USA) using the KAPA SYBR FAST qPCR Master Mix Kit to determine the levels of specific mRNAs. Data were normalized to β-actin mRNA levels, and the expression levels were compared to those of the control (water) group. Oligonucleotide primer sequences used in this study are listed in Table 1.

### 4.7. Western Blot Analysis

Tissue samples were homogenized with the extraction buffer containing 25 mM Tris-HCl (pH 8.0), 1 mM EDTA, 0.5 mM dithiothreitol, 10 mM MgCl_2_, 0.25 mM sucrose, 50 mM sodium fluoride, and 1% (*w/v*) protease inhibitor and centrifuged at 3000 rpm at 4 °C for 10 min. The supernatant protein content was determined using the Bradford assay. For the analysis of L6 cells, cells were washed with ice-cold phosphate-buffered saline and lysed with the RIPA buffer (1x TBS pH 7.4, 0.5% deoxycholic acid, 0.1% sodium deodecyl sulfate [SDS], 1% NP-40, 1 mM PMSF, 1 mM Na_3_VO_4_, 10 mM NaF, and protease inhibitors). After centrifugation, the supernatants were used for Western blot analysis. Total proteins (aliquots containing 30 μg protein) were separated by SDS-polyacrylamide gel electrophoresis (PAGE) using 5–20% e-PAGEL (ATTO Corporation, Tokyo, Japan) or a handmade 10–15% polyacrylamide gel and transferred onto an Immobilon-P membrane (Merck KGaA, Darmstadt, Germany). After blocking with Bullet Blocking One, the membranes were incubated with primary antibodies overnight at 4 °C, washed thrice with Tris-buffered saline with Tween-20 (TBST), incubated with horseradish peroxidase-conjugated secondary antibodies for 60 min, and washed thrice with TBST. Chemiluminescence reaction was performed for 5 min with ImmunoStar LD (Fujifilm Wako Pure Chemical Industries), according to the manufacturer’s protocol. The chemiluminescent signals were visualized and quantified using ImageQuant LAS-4000 and Multi Gauge V3.2 analyzing software (Fujifilm, Tokyo, Japan).

### 4.8. Mitochondrial DNA Analysis

Genomic DNA was extracted from the muscles of rats and L6 cells. The content of mtDNA was analyzed by measuring the relative copy number of the mitochondrial encoded gene, mitochondrial NADH dehydrogenase 1 (*Mt-Nd1*), and nuclear DNA encoded gene, β-actin (*Actb*), by quantitative real-time PCR.

### 4.9. Intracellular Calcium Measurements

Intracellular calcium concentrations were measured by detecting the fluorescence of cells treated with a calcium-sensitive indicator, Fluo-4 AM [54]. L6 cells harvested 10 d after differentiation were replated in a 96-well plate (Iwaki, Tokyo, Japan) at 1.5 × 104 cells/well for 24 h. Subsequently, the Ca^2+^ levels were determined using a Calcium Kit II-Fluo 4 (Dojindo, Kumamoto, Japan) using Powerscan HT (BioTek, VT, USA). Briefly, cells were washed twice with non-serum medium containing 2.5 mM probenecid 24 h after replating. The cells were incubated with 4 μg/mL Fluo-4 AM and 0.025% (*w/v*) pluronic F-127 for 30 min in the dark at 37 °C. After washing twice with non-serum medium, cells were measured using a Powerscan HT instrument with an excitation band of 485/20 nm, and fluorescence intensity was measured at 528/20 nm. Baseline signals (F0) were recorded 5 min before the addition of each stimulus. Continuous fluorescence measurements were performed for 20 min. The results are shown as F/F0 ratios after background subtraction, where F is the fluorescence signal intensity and F0 is the baseline intensity, as calculated from the average of five frames before stimulus application [54].

### 4.10. Statistical Analyses

Data are shown as the mean ± standard error. Results were analyzed using unpaired one-way analysis of variance (ANOVA), followed by Dunnett’s multiple comparisons test for animal experiments and by the Tukey test for experiments with L6 cells for multiple comparisons. For the analysis of [Ca^2+^]i measurements, peaks (maximum F/F0 ratio within 20 min after stimulus) were compared using one-way ANOVA, followed by the Tukey test for multiple comparisons. Groups without the same letter of the alphabet represent significant differences. Statistical significance was set at *p* < 0.05.

All statistical analyses were performed using a statistical database software (SPSS Statistics 27.0 software for Microsoft Windows; IBM, Chicago, IL, USA).

## Figures and Tables

**Table 1:** List of sequences of PCR primers used in this study.

Gene	Direction	Primer Sequence
β-actin (*Actb*)	Forward	5′-GGAGATTACTGCCCTGGCTCCTA-3′
	Reverse	5′-GACTCATCGTACTCCTGCTTGCTG-3′
MEF2A (*Mef2a*)	Forward	5′-ATGAGAGGAACCGACAGGTG-3′
	Reverse	5′-TATCCGAGTTCGTCCTGCTT-3′
PGC-1α (*Ppargc1a*)	Forward	5′-GACCCCAGAGTCACCAAATGA-3′
	Reverse	5′-GGCCTGCAGTTCCAGAGAGT-3′
Succinate dehydrogenase (*Sdha*)	Forward	5′-TGGGGCGACTCGTGGCTTTC-3′
	Reverse	5′-CCCCGCCTGCACCTACAACC-3′
Cytochrome C, somatic (*Cycs*)	Forward	5′-AGCGGGACGTCTCCCTAAGA-3′
	Reverse	5′-CTTCCGCCCAAACAGACCA-3′
Myoglobin (*Mb*)	Forward	5′-CTAACAGCCGGCCTACACTC-3′
	Reverse	5′-CGTGCTTCTTCAGGTCCTCT-3′
GLUT4 (*Slc2a4*)	Forward	5′-GGGCGATTTCTCCCACATAC-3′
	Reverse	5′-CTCATGGGCCTAGCCAATG-3′
TauT (*Slc6a6*)	Forward	5′-CAGTGCCACAGCCTCTTCAG-3′
	Reverse	5′-CTTGCTGGACCACTTCTCCC-3′
Mitochondrial NADH dehydrogenase 1 (*Mt-Nd1*)	Forward	5′-CTCCCTATTCGGAGCCCTAC-3′
	Reverse	5′-ATTTGTTTCTGCTAGGGTTG-3′

## Data Availability

Not applicable.
